# Electroantennographic and Behavioral Evidence Identifies Carvone and IR3535 as Biting Deterrents Against *Rhodnius prolixus* (Stål, 1872)

**DOI:** 10.3390/insects17080829

**Published:** 2026-08-10

**Authors:** Edwin Rodolfo Escobar-Olarte, Gustavo Adolfo Rincón, María Fernanda Vidal, Ruth Mariela Castillo, Agustín Góngora, Sandra Carolina Montaño-Contreras, María Carolina Velásquez-Martínez, Jonny Edward Duque

**Affiliations:** 1 Centro de Investigaciones en Enfermedades Tropicales—CINTROP, Facultad de Salud, Escuela de Medicina, Departamento de Ciencias Básicas, Universidad Industrial de Santander, Guatiguará Technology and Research Park, Km 2 Vía El Refugio, Piedecuesta 681018, Santander, Colombia; edwinstentor@gmail.com (E.R.E.-O.); adolfo.3.8@hotmail.com (G.A.R.); mafevidal.0391@gmail.com (M.F.V.); idiobionte@gmail.com (R.M.C.); 2 Grupo de Investigación GIRGA, Facultad de Ciencias Agropecuarias y Recursos Naturales, Universidad de los Llanos, Villavicencio 1745, Meta, Colombia; agongora@unillanos.edu.co; 3 Grupo de Investigación GYAS, Facultad de Ciencias de la Salud, Universidad de los Llanos, Villavicencio 500001, Meta, Colombia; smontano@unillanos.edu.co; 4 Grupo de Investigación en Neurociencias y Comportamiento UIS-UPB, Facultad de Salud, Escuela de Medicina, Departamento de Ciencias Básicas, Universidad Industrial de Santander, Bucaramanga 680002, Santander, Colombia

**Keywords:** olfactory response, biting deterrence, protection against biting, insect behavior, screening system

## Abstract

Electroantennography (EAG) is a valuable approach for monitoring insect antennal responses to insecticidal and test compounds and may provide a preliminary screening method for identifying compounds with potential activity against medically important insect vectors. The objective of this study was to evaluate the EAG responses of *Rhodnius prolixus* to different xenobiotics and to compare these electrophysiological responses with host approach and protection against biting. Antennae from adult triatomines subjected to prolonged fasting (≥30 days) were exposed to the tested compounds. In parallel, live-bait behavioral bioassays were conducted using *Gallus gallus* and a newly designed laboratory device. Exposure to IR3535 and carvone produced a greater than 60% reduction in ammonia-induced EAG amplitude, consistent with the protection times observed in the behavioral assays (135.6 ± 43.29 min and 108.0 ± 26.33 min, respectively). These findings indicate that IR3535 and carvone alter antennal responses to ammonia and provide prolonged protection against *R. prolixus* bites. Because neither compound significantly delayed host approach, the observed behavioral effect was more consistent with biting deterrence than with demonstrated spatial repellency. EAG may therefore serve as a preliminary complementary screening approach, although its ability to identify compounds that interfere with hematophagous feeding requires further validation using larger and chemically diverse compound libraries.

## Simple Summary

Chagas disease is a major public health problem in Latin America and is transmitted by blood-feeding insects known as triatomines. Current control strategies rely mainly on insecticides; however, the emergence of resistant populations highlights the need for complementary approaches that reduce human–vector contact. In this study, we evaluated the responses of the kissing bug *Rhodnius prolixus* to different chemical compounds using electroantennography, which measures antennal responses to odor stimuli, and a live-bait behavioral assay. IR3535 and carvone markedly reduced ammonia-induced antennal responses and provided prolonged protection against biting. However, neither compound significantly delayed host approach, indicating that their behavioral effect under the experimental conditions was more consistent with biting deterrence than with demonstrated spatial repellency. These findings support the complementary use of electrophysiological and behavioral methods to identify compounds that may interfere with hematophagous feeding.

## 1. Introduction

Chagas disease (CD) constitutes a significant public health problem in Latin America. It is caused by the flagellated protozoan *Trypanosoma cruzi*, transmitted mainly through contact with the feces of hematophagous insect vectors, commonly known as kissing bugs. These insects belong taxonomically to the family Reduviidae, subfamily Triatominae, comprising 158 species across 19 genera [1,2,3]. Because there is no single model of treatment, the most effective strategy to reduce its vector-borne incidence relies on the joint implementation of at least three preventive measures: housing improvement in affected areas, environmental education of communities in risk zones, and vector control through the use of focalized insecticide applications, taking into account the insect’s synanthropic behavior. This latter method is considered the most effective strategy to decrease disease incidence, as reducing triatomine populations interrupts the transmission cycle. In line with this, commercial vector control products are mainly based on pyrethroids such as deltamethrin and cypermethrin. However, the intensive and prolonged use of these compounds over the last decades has led to the emergence of insect populations resistant to their active ingredients [4,5,6].

Chagas disease remains a major public health concern in the Americas, where millions of people are infected, and tens of thousands of new infections continue to occur annually. However, the true burden of the disease remains uncertain due to its often asymptomatic course, limited diagnostic access, underreporting, and differences across national surveillance systems [1,7]. It is paradoxical that repellent molecules have not been widely considered for their potential to protect against vector-borne transmission of this zoonotic disease. Active ingredients such as DEET, picaridin, and IR3535, as well as natural compounds like citronella (*Cymbopogon* spp.), which are regarded as broad-spectrum repellents against hematophagous insects, have been questioned regarding their effectiveness against triatomines due to their limited protection and the lack of comparable evidence on host protection times [8,9,10,11]. A particularly noteworthy aspect is that DEET (N,N-diethyl-m-toluamide), a synthetic molecule considered the “gold standard” for protection against hematophagous insects such as *Aedes aegypti* (Linnaeus, 1762), is notably ineffective against *Rhodnius prolixus* (Stål, 1872). For this reason, it is essential to explore new repellent alternatives and investigate their modes and mechanisms of action further [11,12,13].

Given the urgent need to detect repellent molecules in triatomines and the lack of standardized methods to effectively discriminate compounds with this effect, the insect peripheral nervous system has been proposed as a promising research alternative for the rational design of repellents. Specifically, studies have focused on proteins of the insect olfactory system, such as odorant-binding proteins (OBPs) and odorant receptors (ORs), which play a crucial role in insect behavior. For this reason, both OBPs and ORs constitute alternative molecular targets for identifying compounds with insecticidal or repellent activity, which can be applied to the control of Chagas disease vectors [14,15,16].

As mentioned previously, few studies have used the insect olfactory system as a behavioral assessment tool to select molecules with insecticidal or repellent activity for vector control and protection against bites. Some studies have conducted repellency bioassays through surface impregnation or toxicity assays based on a trial-and-error approach using different molecules under the hypothesis of insecticidal action [17]. However, to date, the responses of the triatomine olfactory system to insecticidal and repellent molecules have not been evaluated using electrophysiological techniques [18,19]. The only existing report of this methodology concerns studies conducted on *Ae. aegypti*, focusing on natural compounds with repellent activity [20]. Therefore, the present study aimed to evaluate the electroantennographic responses of *R. prolixus* to xenobiotics with potential insecticidal and/or bite-protective activity and to compare these responses with behavioral outcomes measured in a live-bait assay. We hypothesized that compounds capable of reducing ammonia-induced EAG responses would provide measurable protection against biting in vivo. We hypothesized that compounds capable of reducing ammonia-induced EAG responses would exhibit measurable behavioral repellency in vivo.

## 2. Materials and Methods

### 2.1. Biological Material

Adult specimens of *R. prolixus* from a colony maintained for more than ten years in the insectary of CINTROP-UIS were used in this study. The insects were maintained in rearing rooms under controlled conditions of 26 ± 3 °C, 75 ± 4% relative humidity, and a 12:12 h light–dark photoperiod. Fifth-instar nymphs were isolated and blood-fed on chickens (*Gallus gallus*) to promote molting to the adult stage. Following adult emergence, the insects were maintained without feeding for approximately 30 days before the experiments; therefore, adults were approximately 30 days post-emergence at the time of testing. The fasting period was used to standardize the insects’ physiological condition before the electroantennographic and behavioral assays and was not considered necessary for antennal responsiveness [11]. Males and females were not separated and were randomly assigned to the experimental groups, and the proportion of each sex was not recorded. Mating status was not individually determined. All electroantennographic and behavioral assays were conducted during the photophase under the controlled environmental conditions described above.

### 2.2. Molecules

**Figure 1:**
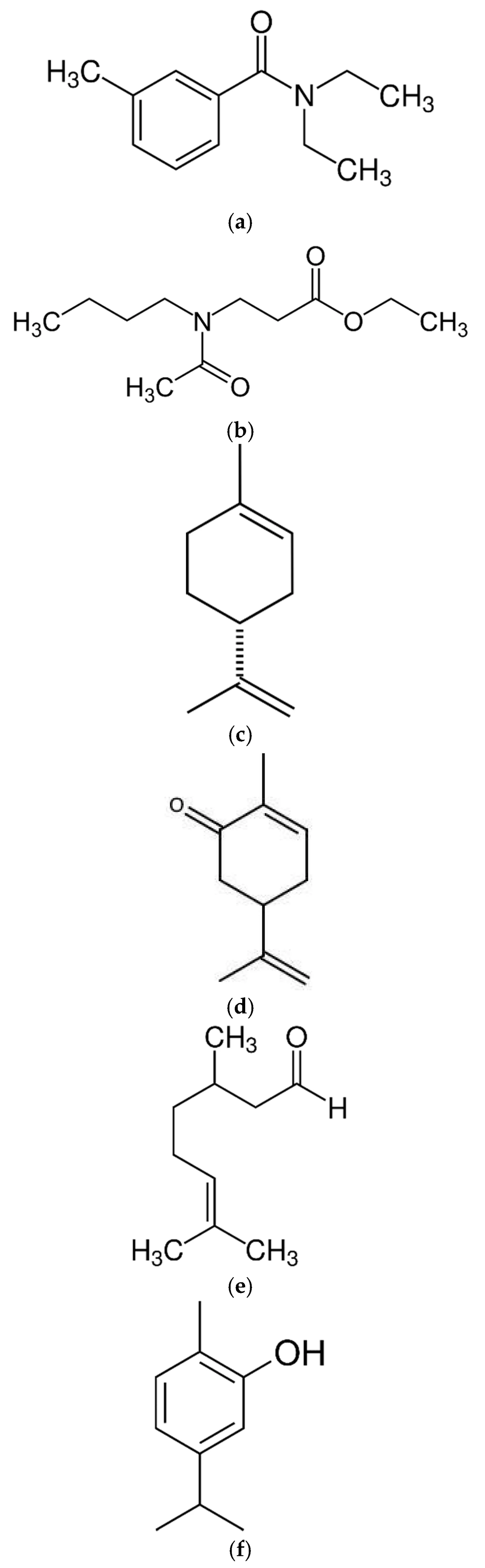
Test compounds used in the experiments: (**a**) DEET, (**b**) IR3535, (**c**) limonene, (**d**) carvone, (**e**) carvacrol, and (**f**) citronellal.

All compounds used in this study were purchased from Merck and were of analytical grade. DEET and IR3535 were included as reference broad-spectrum insect repellents [8,10,20], whereas a commercial ammonium hydroxide solution containing 25% NH_3_ was used as a host-associated olfactory stimulus because behavioral studies have shown that it elicits attraction and oriented locomotor responses in triatomines, including *Rhodnius prolixus* and *Triatoma infestans* [21,22]. Limonene, carvone, carvacrol, and citronellal were selected as test compounds because they are major constituents of essential oils previously reported to have repellent activity against hematophagous insects [20,23,24]. Acetone (99.98%, Merck, Darmstadt, Germany) was used as the solvent control in both bioassays (Figure 1).

### 2.3. Insect Mounting for Electroantennographic Characterization

**Figure 2:**
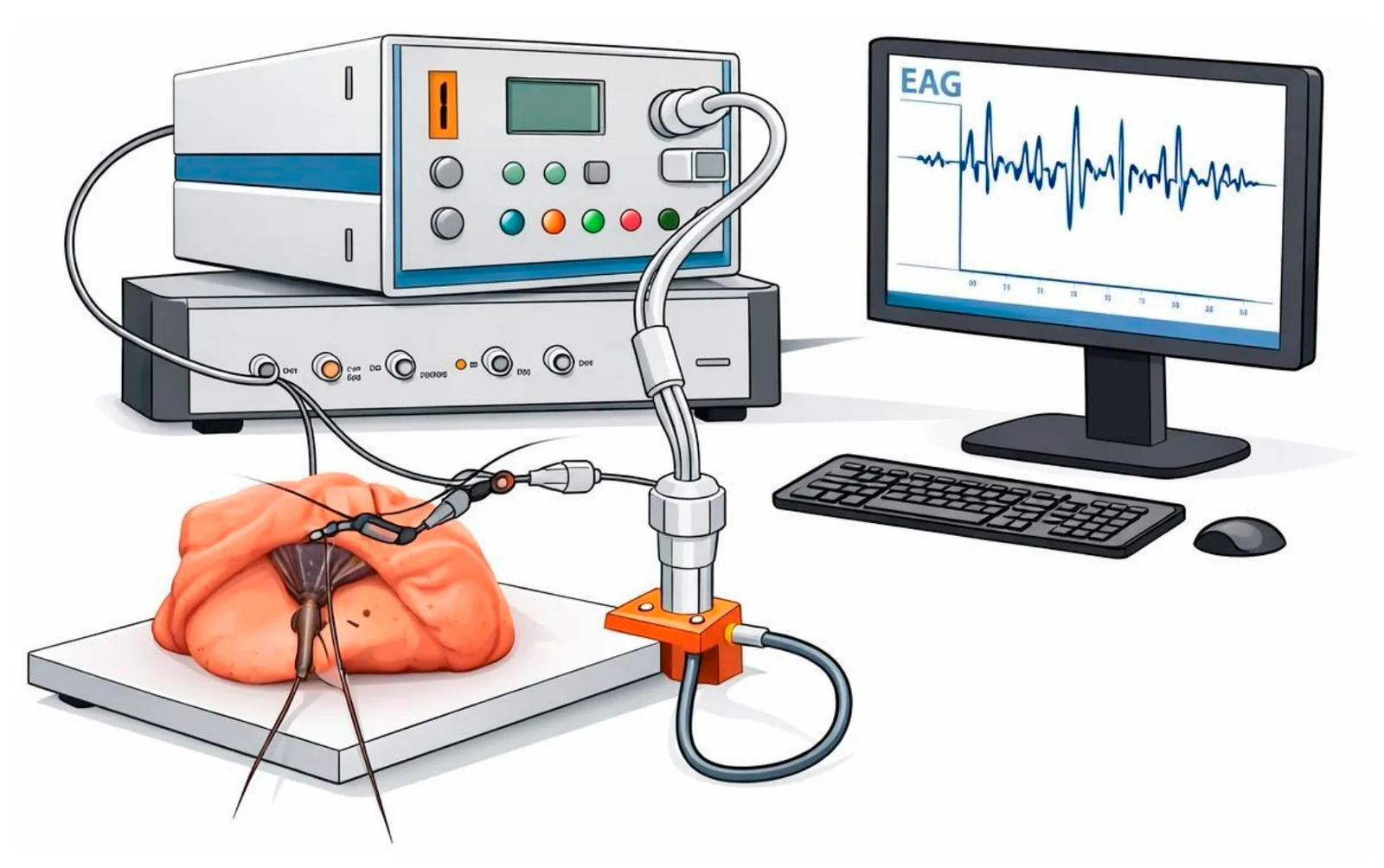
Electroantennography (EAG) experimental setup used to evaluate the olfactory responses of *Rhodnius prolixus* to volatile compounds. The insect was immobilized using modeling clay, leaving the head and antennae exposed for electrophysiological recordings. A recording electrode was inserted into the antenna, while a reference electrode was positioned in the eye region. Volatile stimuli were delivered through a controlled airflow system directed toward the antenna. Electrical signals generated by the antennal response were amplified with an EAG amplifier and recorded using a data acquisition system, enabling visualization and analysis of the electroantennographic signals.

Adult *R. prolixus* individuals subjected to a 30-day programmed fasting period were used to evaluate the electroantennographic (EAG) response of the antennae upon exposure to the test molecules. Each insect was carefully positioned on a wooden platform and immobilized with modeling clay, leaving the antennae and eyes exposed (Figure 2). The mounted specimen was placed under a stereomicroscope (Leica EZ4, Wetzlar, Germany) to facilitate the insertion of two tungsten microelectrodes (0.25 mm, Sigma-Aldrich, St. Louis, MO, USA): one serving as the recording electrode and the other as the reference. Before recordings, the electrodes were electrolytically sharpened by repeatedly immersing their tips in a 1 M sodium hydroxide (NaOH) solution (Sigma-Aldrich) at 3–5 V until the tip diameter reached approximately 0.05–1 mm, following the procedure described by Olsson and Hansson [25]. The reference electrode was inserted into the exposed eye, while the recording electrode was positioned at the base of the antennal pedicel. EAG signals were recorded two minutes after the insect was fixed to the mounting platform, amplified (1000×; Universal Single Probe, Type PRS-1, Syntech, Germany), digitized (IDAC-4, Syntech, Germany), visualized, and analyzed using Autospike software (Syntech, Germany) as previously described by [25].

### 2.4. Olfactory Stimulation and Electroantennographic Recording

Filter paper squares (0.5 × 0.5 cm, Whatman No. 1, Maidstone, UK) were impregnated with 150 µL of each test solution prepared in acetone at 30%, 60%, and 90% (*v*/*v*), corresponding to nominal compound volumes of 45, 90, and 135 µL, respectively. The compounds were prepared and applied volumetrically, and the corresponding masses were not recorded during the experiments. The impregnated filter papers were allowed to dry for one minute to permit solvent evaporation [11,12]. Each impregnated square was then placed inside a glass test tube with a lateral outlet connected to the airflow controller. Pulses of air were delivered through the tube to the insect preparation using a controlled-airflow pump (Stimulus Controller Unit, Type CS-55, Syntech, Buchenbach, Germany). The 10-s pulse duration was selected during preliminary standardization of the experimental conditions to ensure stable exposure of the antenna to the test odor throughout the recording period. The airflow pump was programmed to emit a sequence of three air pulses at a flow rate of 25 mL/s, each lasting 10 s, with 2-s intervals between pulses containing the test compounds and 8 s of clean air serving as background airflow. To monitor antennal responsiveness and evaluate whether the stimulation protocol induced a loss of response, NH_3_ was applied as a positive control before acetone stimulation and again at the end of each recording session.

Before and after the sequential application of the test-compound concentrations, an air pulse containing ammonium hydroxide solution with 25% NH_3_ was delivered to ensure the insect remained responsive to stimuli throughout the experiment and to monitor variations in the electroantennographic signal. Each insect represented one independent replicate and was used to evaluate only one test compound. The three concentrations of the assigned compound were applied sequentially to the same insect, and the insect was not subsequently used for any other compound. Electroantennographic recordings were obtained from a single antenna per insect, and the contralateral antenna was not tested. Ammonia was applied at the beginning and end of each recording session, with 8 s of clean background airflow delivered between stimuli. The amplitude of each EAG response was measured relative to the immediately preceding baseline to minimize the effect of gradual signal drift. Independent materials, including test tubes and connecting tubing, were used to evaluate each compound, with one individual per replicate (*n* = 7 independent insects per compound).

### 2.5. Evaluation of Behavioral Responses and Protection Against Biting

To evaluate the behavioral effects of the test compounds and the protection they provided against biting, bioassays previously reported in the literature were used as references [11,12]. However, due to limited ergonomics and handling difficulties associated with those designs, the Medical Entomology Laboratory at CINTROP developed a new device based on the two previously reported models. A series of comparative bioassays was conducted between the two device types to compare the protection-time measurements obtained with the newly designed apparatus and the previously reported device.

### 2.6. CINTROP Device Specifications

**Figure 3:**
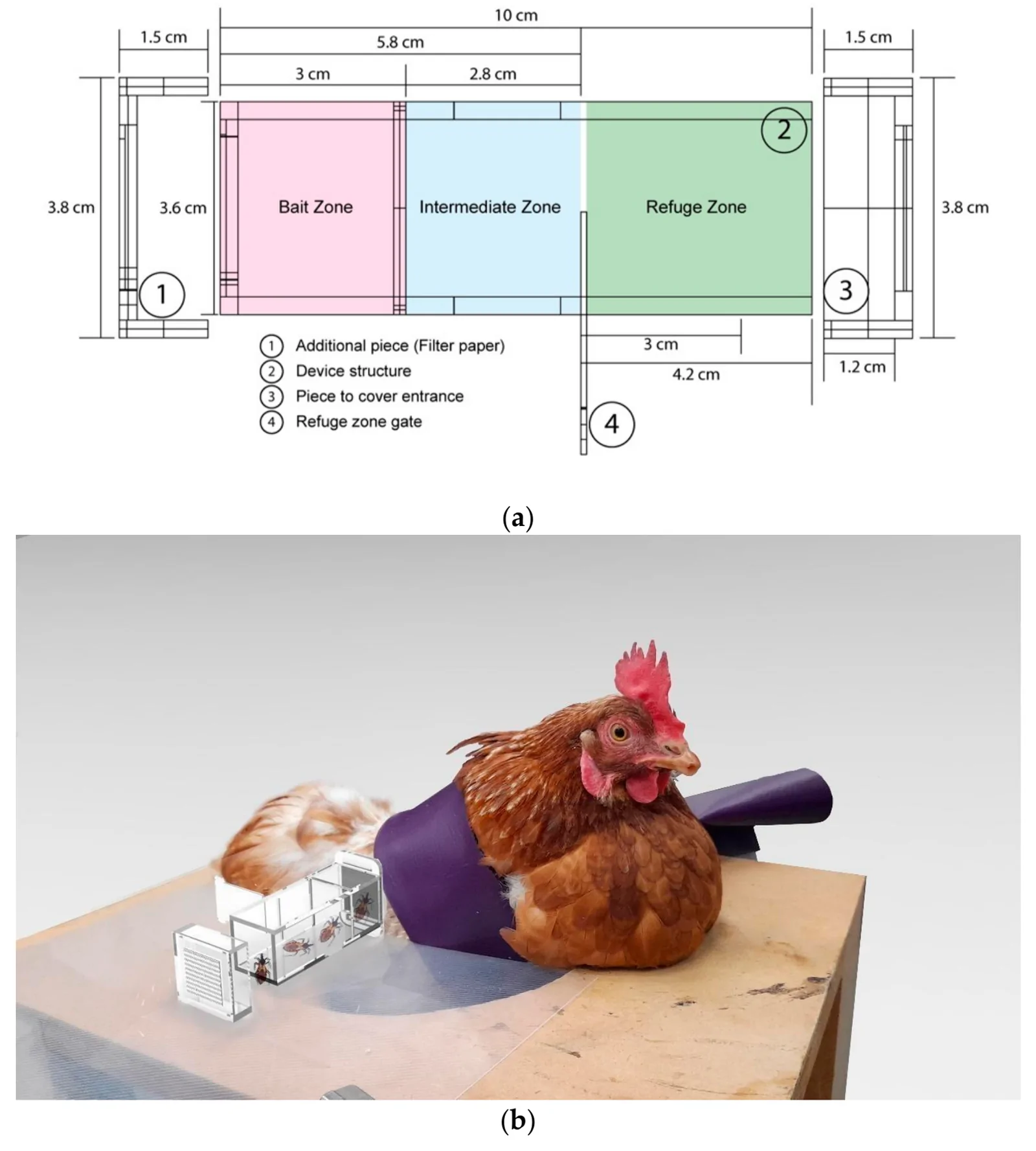
Design specifications of the proposed device for evaluating repellent activity in triatomines. (**a**) Schematic representation; (**b**) Device coupled to the bait.

The device was constructed from 3 mm-thick transparent acrylic sheets. It has a rectangular shape, measuring 10 cm in length and 3.6 cm in width, providing a total contact area of 36 cm^2^ (Figure 3). The proximal end features a mesh and an additional acrylic component that allows the insertion of filter paper, enabling either direct or indirect contact with live bait. Located 3 cm from the mesh, an internal division engraved into the acrylic separates the Bait Zone from the Intermediate Zone. The distal end features a movable gate positioned 3.2 cm from the device’s farthest edge, which defines the Refuge Zone. The Refuge Zone was designed to provide sufficient space to introduce and temporarily retain the insects before the assay. Its length was only slightly greater than that of the Bait Zone (3.2 vs. 3.0 cm) and did not represent a difference in the relative importance of the two zones. The Bait Zone was restricted to the terminal area immediately adjacent to the host because its function was specifically to record host-directed approach. The movable gate separated the Refuge Zone from the Intermediate Zone and prevented insects from moving toward the bait before the assay began.

The apparatus also includes a fixation system for use with live, non-anesthetized bait (e.g., a chicken).

### 2.7. Determination of Host Approach and Protection Against Biting

Both devices (Zermoglio and CINTROP) were evaluated simultaneously using adult chickens (*Gallus gallus*) as live bait and adult *R. prolixus* individuals. At the beginning of each replicate, the insects were placed in the Refuge Zone while the movable gate remained closed. The gate prevented movement toward the bait during insect introduction and the initial acclimation period. At the start of the observation period, the gate was opened and remained open, allowing the insects to move freely through the Refuge, Intermediate, and Bait Zones.

Each bird was topically treated with 150 µL of the test solution applied to one side of the abdomen, beneath the wings. This volume was selected because it provided uniform coverage of the defined treatment area without dripping or spreading beyond the intended region. The dose was not normalized to the bird’s total body surface area; instead, a fixed volume was applied to the same anatomical region across all assays to standardize exposure conditions. Initially, the commercial repellent IR3535 was tested at a concentration of 90%. The behavioral response of the insects was assessed using both the plastic device described by Zermoglio et al. [12] and the CINTROP prototype. For each test compound, 15 insects were evaluated individually per experimental day; the insects were not tested simultaneously as a group. Each insect represented an individual behavioral observation. The complete assay was conducted on at least three different days, using a different live bait each day; each experimental day and corresponding live bait constituted an independent biological replicate. Acetone controls were evaluated under the same experimental conditions.

Protection against biting (PC) and approach to the bait (AC) were recorded from 0 to 150 min, including 30-min resting intervals to ensure animal welfare. The approach to the bait was measured as the elapsed time from the start of the assay to the first host-directed feeding attempt. A feeding attempt was identified when an insect entered the Bait Zone and extended its proboscis toward the host, regardless of whether it penetrated the skin. Protection against biting was operationally defined as the absence of successful proboscis insertion and feeding initiation on the treated area. The assay was terminated upon the first confirmed bite; therefore, protection time was defined as the elapsed time from the application of the test compound to the first confirmed bite or to the maximum observation time of 150 min. Subsequently, DEET, limonene, carvone, carvacrol, and citronellal were evaluated at 90%, selected based on previous studies in triatomines and the initial concentration-screening assays [11,12]. This common screening concentration enabled direct comparison among compounds, although the assays were not designed to establish concentration–response relationships.

### 2.8. Ethics Statement

All experimental procedures were approved by the Ethics Committee “Comité de ética en investigación científica CEINCI” (Scientific Research Ethics Committee at the Industrial University of Santander, acronym in Spanish CEINCI), No. 08, 11 May 2018. Furthermore, the study was carried out in compliance with the ARRIVE guidelines and in accordance with the 1989 Colombian Law 84 (Chapter IV, Art. 23–26) and Resolution 8430 (1993, Title IV, Art. 83–93), which regulate animal research in Colombia.

### 2.9. Statistical Analysis

Electroantennographic (EAG) responses were calculated as the mean maximum amplitude (mV) recorded for each administered air pulse. All behavioral and EAG data were tested for normality using the Kolmogorov–Smirnov test. Analysis of variance was then performed to compare maximum EAG responses among the different compounds (ANOVA or Kruskal–Wallis, depending on data normality), followed by multiple comparison tests (Tukey’s or Dunn’s, depending on the normality results). For the live-bait behavioral bioassays, protection times obtained with the Zermoglio and CINTROP devices were compared using the Mann–Whitney U test. Host-approach times among treatments were analyzed using one-way ANOVA. Protection times among the tested compounds were compared using the Kruskal–Wallis test, followed by Dunn’s post hoc multiple-comparison test when the overall difference was statistically significant. Only *p*-values < 0.05 were considered statistically significant.

## 3. Results

### 3.1. EAG Records of Xenobiotic Compounds 

**Figure 4:**
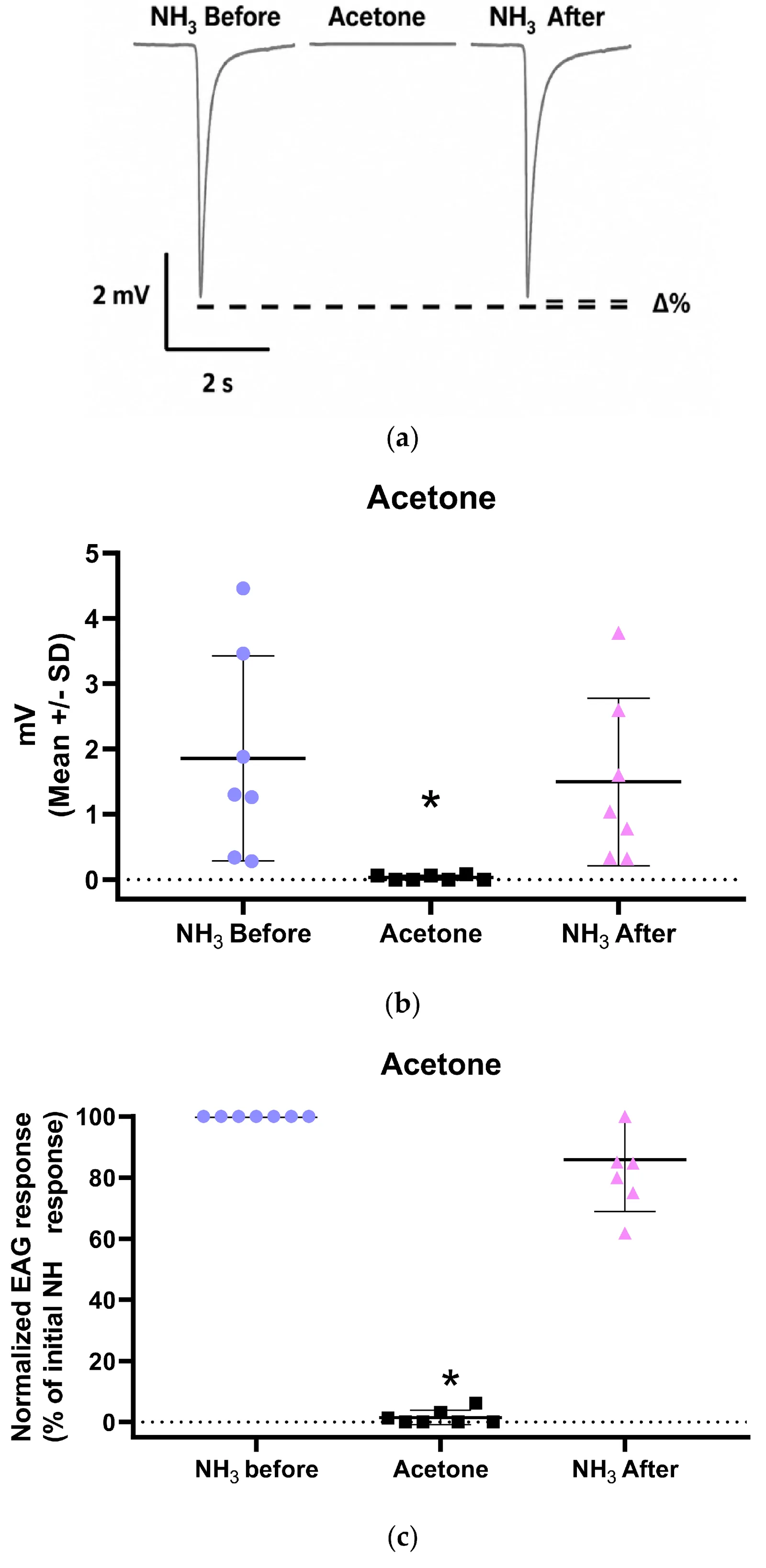
Electroantennographic (EAG) responses to acetone (negative control), with pre- and post-exposure stimulation using a 25% NH_3_ ammonium hydroxide solution. (**a**) Representative EAG responses. (**b**) EAG amplitudes expressed in millivolts (mV; mean ± standard deviation). (**c**) Normalized EAG responses, with the initial ammonia-induced response defined as 100% and the acetone-induced and final ammonia-induced responses expressed relative to this reference (mean ± standard deviation). The Kruskal–Wallis test showed an overall significant difference among the experimental groups (*H*(3, *N* = 21) = 17.44, *p* < 0.05). However, Dunn’s post hoc test showed no statistically significant difference between the NH_3_-induced EAG responses recorded before and after acetone exposure. Significant pairwise differences were observed between the acetone response and the NH_3_ responses. * Indicates a statistically significant difference from all treatments (*p* < 0.05).

To evaluate the olfactory response of *R. prolixus*, electroantennographic (EAG) signals were recorded after exposure to the tested xenobiotic compounds. Each insect exhibited a distinct EAG pattern, resulting in a wide range of amplitudes among individuals. This inter-individual variability may reflect natural differences in antennal sensitivity and physiological condition, as well as minor technical variations associated with antennal preparation and electrode placement. Consequently, data normalization was performed using the initial and final ammonia amplitude responses from all tested compounds, taking the initial ammonia response as the 100% reference. This normalization allowed compound-induced changes to be expressed relative to each individual’s baseline responsiveness and facilitated comparisons among treatments. A preliminary bioassay was conducted to assess whether acetone, the solvent used for all test compounds, altered antennal responsiveness to ammonia. The results showed no statistically significant differences between the insects’ initial and final ammonia responses, indicating that acetone did not produce a detectable change in ammonia-induced EAG amplitude under the experimental conditions (Figure 4).

The same experimental procedure was then applied to the test compounds, which were dissolved in acetone at concentrations of 90%, 60%, and 30% (*v*/*v*). An initial ammonia recording was taken, followed by exposure to the test compound, and finally, a second ammonia recording was performed. The EAG amplitudes elicited directly by the test compounds were considerably lower than those elicited by ammonia, not exceeding 10% of the ammonia-induced response. For most test compounds, no statistically significant differences were observed between the initial and final ammonia recordings, indicating that exposure did not produce a detectable change in ammonia-induced antennal responsiveness. In contrast, IR3535 and carvone produced marked reductions in the final ammonia-induced EAG response.

**Figure 5:**
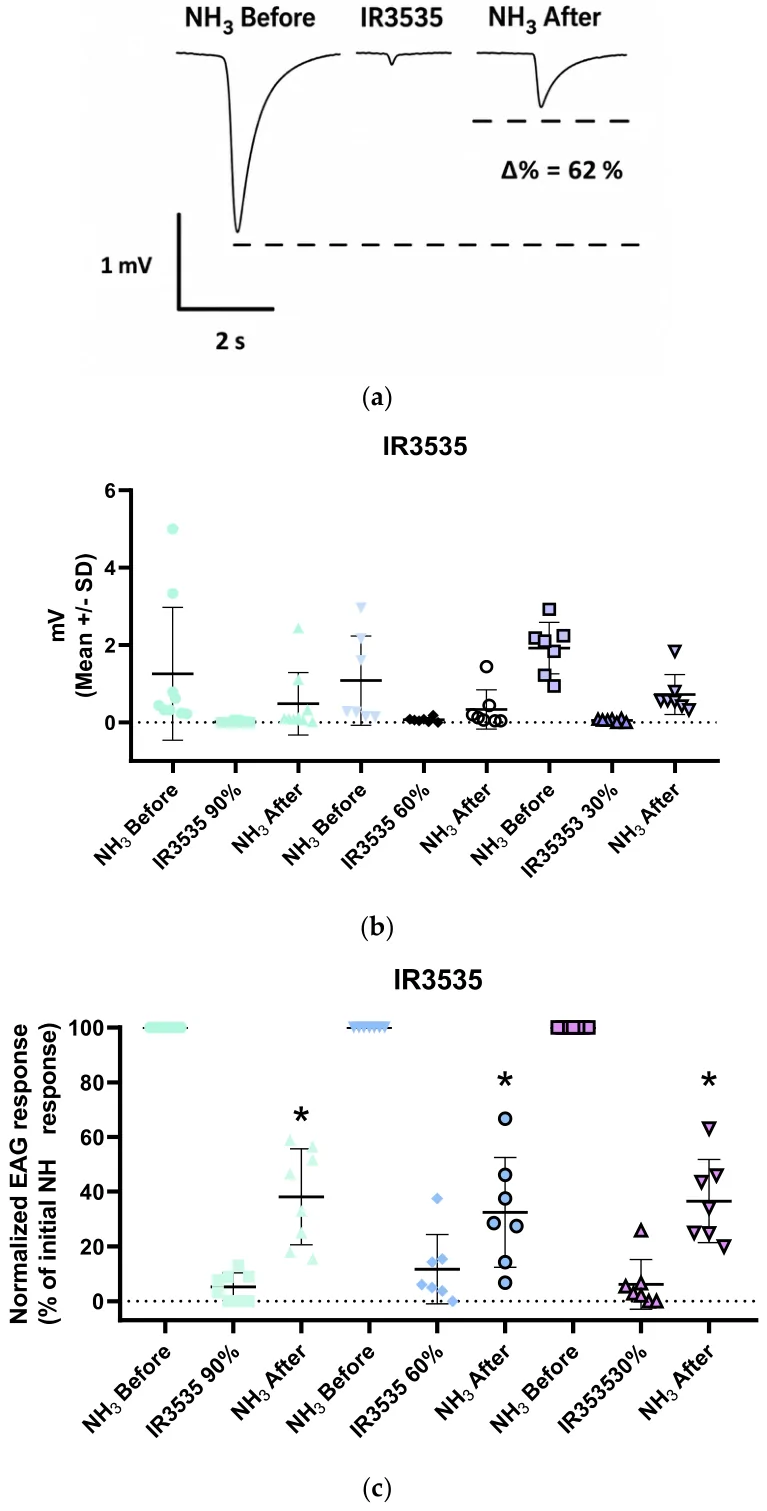
Electroantennographic signals and percentage change in the ammonia response after exposure to IR3535, an ammonium hydroxide solution containing 25% NH_3_. (**a**) Representative electroantennogram (EAG) recordings. (**b**) EAG amplitudes recorded at 30%, 60%, and 90% (mean ± standard deviation), expressed in millivolts (mV). (**c**) Normalized EAG responses at 30%, 60%, and 90% (*v*/*v*), with the initial ammonia-induced response defined as 100% and the IR3535-induced and final ammonia-induced responses expressed relative to this reference (mean ± standard deviation). Seven independent adult insects were evaluated (*n* = 7 per concentration); the same seven insects were sequentially exposed to the three concentrations. An asterisk indicates a statistically significant difference between the ammonia-induced EAG responses recorded before and after IR3535 exposure (Kruskal–Wallis test, *H*(3, *N* = 21) = 17.44, *p* < 0.05; Dunn’s post hoc test).

**Figure 6:**
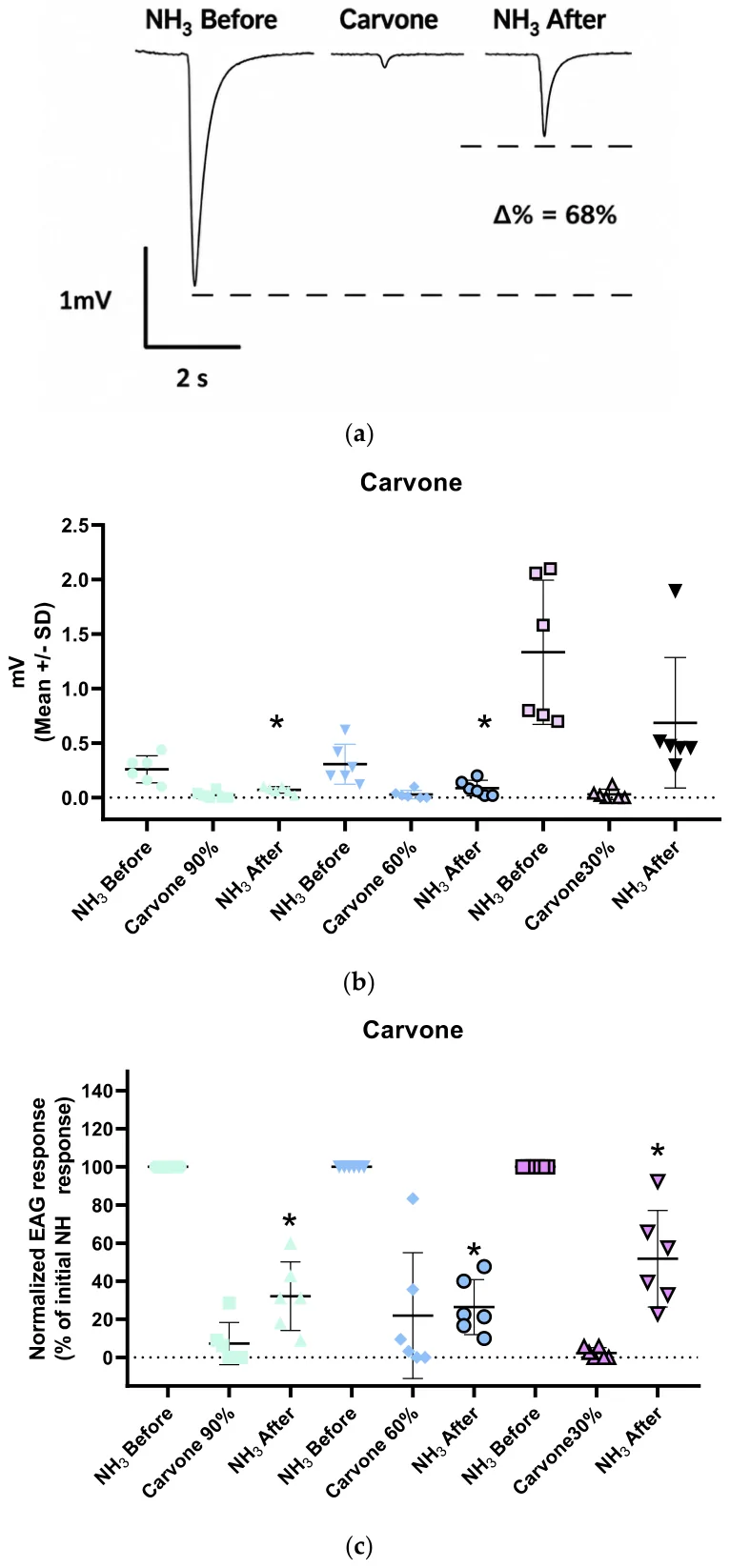
Electroantennographic responses to carvone, with pre- and post-exposure stimulation using an ammonium hydroxide solution containing 25% NH_3_. (**a**) Representative electroantennogram (EAG) responses. (**b**) Electroantennographic signal recordings at three concentrations (90%, 60%, and 30%), expressed in millivolts (mV). (**c**) Normalized EAG responses at 30%, 60%, and 90% (*v*/*v*), with the initial ammonia-induced response defined as 100% and the carvone-induced and final ammonia-induced responses expressed relative to this reference (mean ± standard deviation). Seven independent adult insects were evaluated (*n* = 7); the same insects were sequentially exposed to the three concentrations. An asterisk indicates statistically significant differences between the ammonia-induced EAG signals recorded before and after exposure to carvone (one-way ANOVA, *F*(8, 45) = 37.43, *p* < 0.05; Tukey’s post hoc test).

Among the tested compounds, IR3535 and carvone produced the largest reductions in the ammonia-induced EAG response, with decreases exceeding 60% after compound exposure. For IR3535, the overall analysis revealed significant differences among the experimental groups (Kruskal–Wallis test, *H*(3, *N* = 21) = 17.44, *p* < 0.05), and the corresponding pairwise comparisons were evaluated using Dunn’s post hoc test (Figure 5). Carvone also produced a significant change in the ammonia-induced EAG response. The comparison among the experimental groups was significant (one-way ANOVA, *F*(8, 45) = 37.43, *p* < 0.05), followed by Tukey’s post hoc multiple-comparison test (Figure 6). The remaining compounds did not produce statistically significant changes in the ammonia-induced EAG response following exposure.

### 3.2. Host Approach and Protection Against Biting

#### 3.2.1. Comparison Between CINTROP and Zermoglio Devices

In the comparison between the CINTROP and Zermoglio devices, protection against bites produced by the IR3535 compound at a 90% concentration was evaluated as a parameter of repellent activity. No statistically significant differences in protection time were observed between the two devices used against *R. prolixus* (Table 1). However, statistically significant differences were detected between the repellent compound IR3535 and its control (acetone) (Mann–Whitney U test, *p* < 0.05).

Considering these results and the absence of statistically significant differences among the devices used in the standardization bioassays, the device designed by the CINTROP laboratory was selected for subsequent experiments. The CINTROP device can be securely attached to the live bait, facilitating experimental handling and reducing disturbances caused by host movement. It produced protection-time estimates and variability comparable to those obtained with the Zermoglio device.

#### 3.2.2. Evaluation of Test Compounds for Protection Against Biting

**Figure 7:**
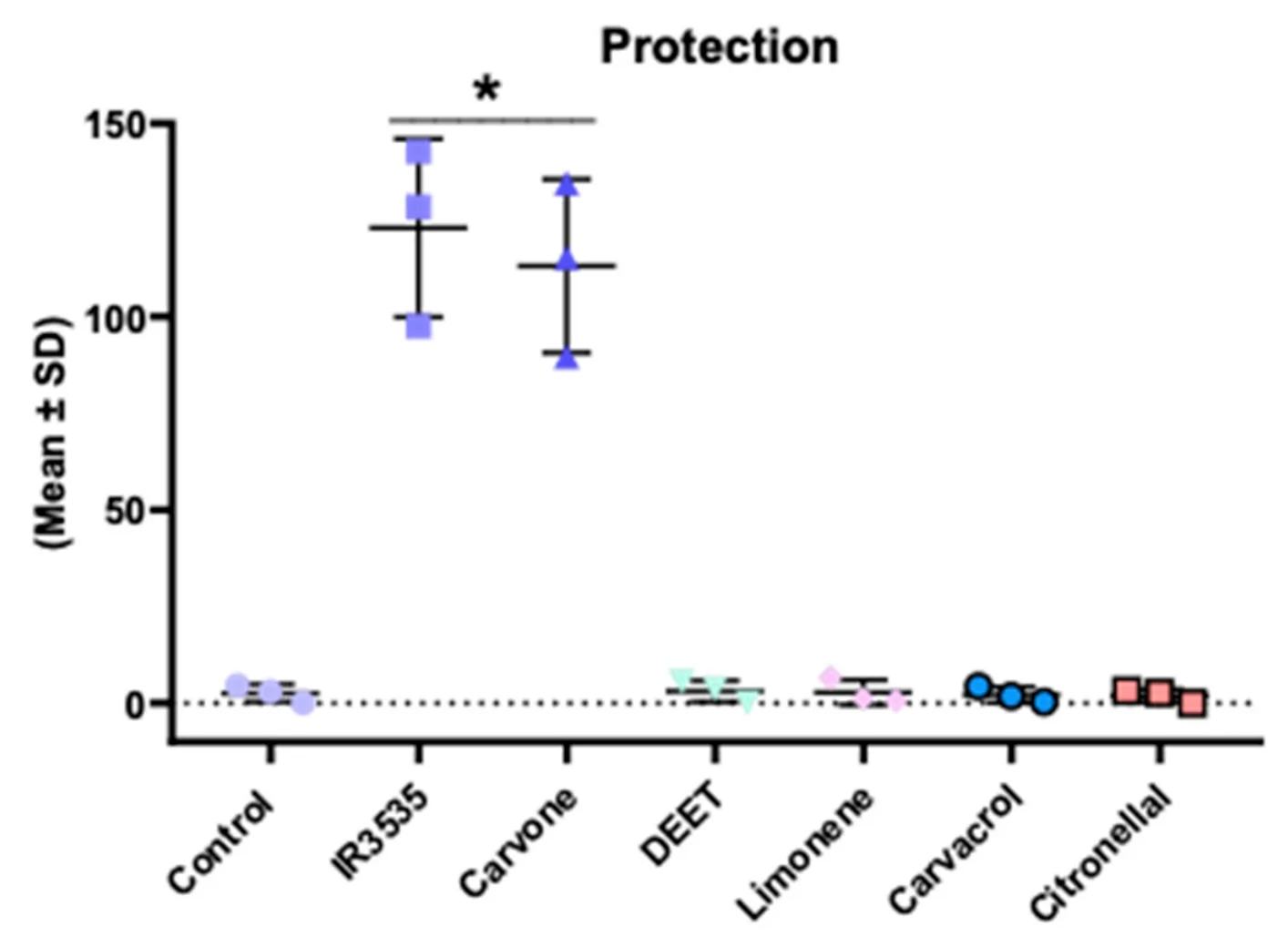
Protection time against *Rhodnius prolixus* biting after application of the solvent control and the test compounds at 90%. Individual symbols represent independent experimental observations, and horizontal lines with error bars indicate the mean ± standard deviation (*n* = 3 independent observations per treatment). IR3535 and carvone did not differ significantly, but both showed significantly longer protection times than the other treatments (*p* < 0.05). * Indicates a statistically significant difference from all treatments, except between IR3535 and carvone (*p* < 0.05).

Two commercial repellents (DEET and IR3535) and four major components of essential oils (limonene, carvone, carvacrol, and citronellal) were evaluated at 90% concentration. Acetone was used as a negative control. IR3535 provided the longest protection against biting, with a protection time against insect bites of 135.6 ± 43.29 min (mean ± standard deviation), followed by carvone, with a mean protection time of 108 ± 26.33 min. Both compounds showed statistically significant differences compared with the other molecules evaluated, with protection lasting at most 5 min (Figure 7).

#### 3.2.3. Approach to the Host

In addition to protection time, host-approach time was recorded as the elapsed time from the beginning of the assay until the first insect entered the Bait Zone after moving through the Intermediate Zone. Turning toward the host or moving only within the Refuge or Intermediate Zones was not considered a host-approach event. Once in the Bait Zone, insects were observed extending their proboscis toward the host. In the presence of IR3535 or carvone, insects could approach the host but subsequently retreated toward the Refuge Zone without producing a successful bite. In the remaining treatments, the host approach was followed by successful skin penetration and visible puncture marks on the chicken skin. Host-approach times are presented in Table 2 and did not differ significantly among treatments (one-way ANOVA, *F*(6, 61) = 1.066, *p* = 0.39).

## 4. Discussion

### 4.1. Electroantennographic Recordings

The present study evaluated the electrophysiological and behavioral responses of *R. prolixus* to a selected group of volatile compounds. IR3535 and carvone showed both a reduction in the ammonia-induced EAG response and prolonged protection against biting under the experimental conditions. Because neither compound significantly delayed host approach, the behavioral effect was more consistent with biting deterrence than with demonstrated spatial repellency. Although no formal correlation analysis was performed, the occurrence of both responses supports the complementary use of EAG recordings and behavioral bioassays for the preliminary evaluation of compounds capable of interfering with hematophagous feeding.

Initially, the olfactory system of insects was stimulated with ammonia (NH_3_), a compound known to play a key role in host-seeking behavior in hematophagous insects and is commonly used as a standard stimulus in electroantennographic (EAG) assays due to its consistent, concentration-dependent responses in *R. prolixus* [18]. As expected, stimulation with ammonia elicited clear electrophysiological responses in the olfactory neurons, with signal amplitudes ranging from 0.26 to 4 mV. These responses reflect activation of ion channels involved in odor reception, with higher voltage amplitudes indicating greater ionic flux across neuronal membranes, resulting in membrane depolarization [26]. However, the responses elicited by the evaluated compounds were considerably lower than those produced by ammonia. Ammonia was therefore used as a reference stimulus to evaluate changes in antennal responsiveness following exposure to the tested compounds.

### 4.2. Electroantennographic Responses to the Test Compounds

The present results indicate that IR3535 and carvone provided prolonged protection against biting and produced significant changes in the ammonia-induced EAG responses of *R. prolixus*. In particular, IR3535 and carvone altered antennal responsiveness, as confirmed by comparing initial and final ammonia recordings, in which a reduction of approximately 60% in signal amplitude was observed at the highest dose. This decrease, together with the absence of a detectable effect on host-approach time, indicates that their behavioral effects were more consistent with biting deterrence than with demonstrated spatial repellency. From a physiological perspective, reductions in EAG amplitude are generally associated with changes in the activity of odorant receptor neurons (ORNs), which play a central role in olfactory perception in hematophagous insects. However, the present results do not demonstrate that the compounds impaired host detection or host orientation.

Similar effects have been described for well-known repellents such as DEET, which can attenuate antennal responses to attractive odors by inhibiting or modulating ORN activity [27]. Other compounds, including picaridin and IR3535, have also been reported to affect olfactory signal transduction in blood-feeding insects through comparable mechanisms [28]. Together, these findings indicate that commercially recognized repellents can modulate peripheral olfactory signaling. However, the reduction in EAG amplitude observed in the present study does not by itself demonstrate disruption of host detection. Instead, the findings highlight the usefulness of EAG recordings as a complementary screening tool for identifying compounds that should subsequently be evaluated for effects on hematophagous feeding and host-oriented behavior.

In the present study, host approach and protection against biting were determined using behavioral bioassays, whereas EAG recordings were used to evaluate changes in ammonia-induced antennal responsiveness. IR3535 and carvone provided prolonged protection against biting, with protection times exceeding 100 min in the live-bait assays, and produced reductions of approximately 60% in ammonia-induced EAG amplitude. In contrast, compounds with protection times shorter than 5 min did not produce statistically significant changes in the ammonia-induced EAG response. These parallel behavioral and electrophysiological findings suggest that EAG recordings may help prioritize candidate compounds for subsequent behavioral evaluation. However, because only six compounds were tested, the present dataset is insufficient to establish the reliability or predictive performance of EAG as a general screening method. Validation using a larger and chemically diverse library, including compounds with and without biting-deterrent activity, will be required to determine whether specific EAG response patterns can reliably identify compounds capable of interfering with hematophagous feeding.

### 4.3. Interpretation of the Behavioral Effect

There is currently no fully standardized live-bait method for assessing protection against triatomine bites. The CINTROP device was developed as an operational modification of the Zermoglio apparatus rather than as a more reproducible or more sensitive behavioral assay. Its rigid acrylic structure can be securely attached to the live host, reduces displacement associated with host movement, permits continuous observation of the insects, and facilitates replacement of treated filter papers during repeated assays. Protection times and variability obtained with the CINTROP and Zermoglio [12] devices were comparable, indicating that the structural modifications did not alter the principal behavioral endpoint.

The present assay primarily measured protection against biting rather than spatial repellency. IR3535 and carvone did not significantly delay host approach, because insects entered the Bait Zone under these treatments. However, they failed to complete a successful bite and were observed moving away from the host after close-range exposure. These findings are therefore more consistent with biting or feeding deterrence than with demonstrated long-range repellency. Because the experimental zones were short, the device may not provide sufficient distance to resolve effects on semiochemical-mediated orientation. Additional olfactometer or motion-tracking experiments will be required to determine whether IR3535 and carvone also produce spatial repellency.

All compounds were compared at 90%, the common concentration selected from the initial concentration-screening assays. IR3535 was initially used to compare the two live-bait devices, whereas DEET was retained as a previously established reference compound. Thus, the use of IR3535 at 90% should not be interpreted as overcoming the need for high concentrations reported for DEET, but as part of a standardized comparative design.

### 4.4. Protection Against Biting Produced by the Test Compounds

Among all the compounds evaluated, only the xenobiotic carvone showed a clear protective effect against insect bites, providing protection times exceeding 100 min. This response was statistically similar to that observed for the reference compound IR3535. In contrast, both compounds showed significant differences compared with the remaining tested molecules, which did not exceed 5 min of protection. These results indicate that only IR3535 and carvone provided prolonged protection against biting under the experimental conditions used in this study. Because neither compound significantly delayed host approach, this effect is more consistent with biting deterrence than with demonstrated spatial repellency [29].

## 5. Conclusions

Overall, the results obtained in this study indicate that carvone and IR3535 produce significant reductions in ammonia-induced EAG responses and provide prolonged protection against biting by *R. prolixus*. Because neither compound significantly delayed host approach, the behavioral effect observed in the present bioassay is more consistent with biting or feeding deterrence than with demonstrated spatial repellency. Integrating electrophysiological recordings with live-bait behavioral bioassays enabled the identification of compounds that exhibited both altered ammonia-induced antennal responses and reduced biting, without establishing a direct causal mechanism between these effects. In addition, the evaluation and validation of the CINTROP device provide a practical methodological alternative for measuring protection time under conditions that better simulate close-range host–vector interactions. Taken together, these findings support the use of combined behavioral and electrophysiological approaches as complementary methods for the preliminary screening of compounds capable of interfering with hematophagous feeding and highlight the potential of such strategies to contribute to the development of non-insecticidal interventions to improve vector control of Chagas disease.

## Figures and Tables

**Table 1:** Comparison of protection time between the Zermoglio and CINTROP devices. Mean protection time against *R. prolixus* bites (min ± SD) using IR3535 at 90% concentration.

Xenobiotics	Zermoglio Protection Time (Min, Mean ± SD)	Cintrop Protection Time (Min, Mean ± SD)
Negative control (Acetone)	12.18 ± 8.68 ^a^	7.46 ± 6.97 ^a^
IR3535 at 90% concentration	118.43 ± 27.34 ^b^	134.45 ± 26.93 ^b^

Identical letters indicate that there are no statistically significant differences between devices (Mann–Whitney U test, U = 2.0, *p* = 0.38); different letters indicate significant differences between the repellent and its control within each device (*p* < 0.05) in *R. prolixus.*

**Table 2:** Host-approach time for the evaluated compounds: IR3535, carvone, DEET, limonene, carvacrol, citronellal, and control (mean ± standard deviation). No statistically significant differences were observed among the bioassays performed (ANOVA, *F*(6,61) = 1.066; *p* = 0.39).

Xenobiotics (90% *v*/*v*)	Host-Approach Time (Min; Mean ± SD)
IR3535	4.9 ± 4.3
Carvone	2.4 ±2.3
DEET	3.0 ± 2.7
Limonene	2.8 ± 3.2
Carvacrol	2.1 ± 2.0
Citronellal	1.9 ± 1.6
Control (Acetone)	2.6 ± 2.2

## Data Availability

The original contributions presented in this study are included in the article. Further inquiries can be directed to the corresponding authors.
